# Robust Off- and Online Separation of Intracellularly Recorded Up and Down Cortical States

**DOI:** 10.1371/journal.pone.0000888

**Published:** 2007-09-12

**Authors:** Yamina Seamari, José A. Narváez, Francisco J. Vico, Daniel Lobo, Maria V. Sanchez-Vives

**Affiliations:** 1 Departamento Fisiología General, Facultad de Medicina, Universidad de Málaga, Málaga, Spain; 2 Departamento Lenguajes y Ciencias de la Computación, Escuela Técnica Superior de Ingeniería Informática, Universidad de Málaga, Málaga, Spain; 3 Instituto de Neurociencias de Alicante, Universidad Miguel Hernández-Consejo Superior de Investigaciones Científicas, Alicante, San Juan de Alicante, Spain; Indiana University, United States of America

## Abstract

**Background:**

The neuronal cortical network generates slow (<1 Hz) spontaneous rhythmic activity that emerges from the recurrent connectivity. This activity occurs during slow wave sleep or anesthesia and also in cortical slices, consisting of alternating up (active, depolarized) and down (silent, hyperpolarized) states. The search for the underlying mechanisms and the possibility of analyzing network dynamics *in vitro* has been subject of numerous studies. This exposes the need for a detailed quantitative analysis of the membrane fluctuating behavior and computerized tools to automatically characterize the occurrence of up and down states.

**Methodology/Principal Findings:**

Intracellular recordings from different areas of the cerebral cortex were obtained from both *in vitro* and *in vivo* preparations during slow oscillations. A method that separates up and down states recorded intracellularly is defined and analyzed here. The method exploits the crossover of moving averages, such that transitions between up and down membrane regimes can be anticipated based on recent and past voltage dynamics. We demonstrate experimentally the utility and performance of this method both offline and online, the online use allowing to trigger stimulation or other events in the desired period of the rhythm. This technique is compared with a histogram-based approach that separates the states by establishing one or two discriminating membrane potential levels. The robustness of the method presented here is tested on data that departs from highly regular alternating up and down states.

**Conclusions/Significance:**

We define a simple method to detect cortical states that can be applied in real time for offline processing of large amounts of recorded data on conventional computers. Also, the online detection of up and down states will facilitate the study of cortical dynamics. An open-source MATLAB® toolbox, and Spike 2®-compatible version are made freely available.

## Introduction

The slow (<1 Hz) oscillation, as described in cortical neurons of naturally sleeping [1], [2] and anesthetized [1], [3]–[5] cats, as well as in the sleep EEG and magnetoencephalograms of humans [6]–[8] comprises a periodic fluctuation between a hyperpolarized membrane potential or down state (characterized by the absence of network activity), and a depolarized membrane potential, or up state (where action potentials use to occur).

The slow oscillation is cortically generated [9] and takes place as a stable synchronous network event as demonstrated by multiple intra- and extracellular recordings in the intact brain [10]–[12]. Its generation by the cortical network is backed by the fact that it is also generated in deafferented cortical slabs [13] and in cortical slices maintained *in vitro* [14]. A large number of studies have been published in recent years dealing with the cellular and network mechanisms underlying this slow rhythm and other related aspects, such as the effect of up and down states on synaptic transmission and excitability [15]–[21].

In order to understand the cellular and network properties that modulate slow membrane potential fluctuations, it is often required to detect, separate and quantify the up and down states for further detailed data analysis. To achieve this processing of intracellularly recorded membrane potential fluctuations some methods deal with the data in a manual fashion, while others implement basic automated procedures.

Metherate and Ashe (1993) [22] first carried out the quantification of the two-state behavior based on the membrane potential distribution. That graphical tool operates on the characteristic bimodal distribution of the membrane potential, best fitted to a dual Gaussian function, and has been extensively used since then [14], [19], [23]–[33]. A peak at the hyperpolarized membrane potential values identifies the down state, separated from the depolarized up state by a well-defined central valley, indicative of fast transitions between the two states. Recently, a moving average of the membrane potential and its standard deviation (SD) has been presented [12] to separate the two states. In this case the down state presents a sharp peak at hyperpolarized potentials with low SD values, while the up state shows a broader hill at more depolarized potentials and higher SD values. A different approach based on the spectral difference of the LFP (local field potential) signal has been recently proposed to distinguish between up and down states [34]. This method also relies on the bimodal distribution of the membrane potential.

The basic assumption underlying the approaches based on the bimodal distribution of the membrane potential is that the proportion of the area of the histogram under each of the peaks represents the proportion of time spent in each state, and consequently the mode of each peak is the preferred membrane potential in each state. While this is true for very stable recordings, data is typically affected by fluctuating electrical and physiological conditions.

According to this property, these approaches proceed by performing certain measurements on the biphasic histogram. A basic operation is to determine the threshold potential that delimits both states. This is obtained by computing the modes of the distributions (or, alternatively, visually identifying the peaks) and finding either the potential associated with the lowest bar between them, or the midpoint between the peaks if a broad valley separates them [35]. More reliable transitions can be performed by setting two thresholds, e.g., at one fourth and three fourths of the distance between the peaks [23]. The areas separated by these delimiting values are a good estimation of the time spent in each mode.

Despite the simplicity and popularity of the histogram-based methods, there are some disadvantages related to its use:

1. The intracellular membrane potential recordings must be stable over the time window used to compute the histogram. However, this ideal scenario is frequently complicated by membrane potential drift, changes in the electrode seal, movement artifacts (e.g. respiratory movements, heartbeat) or other factors, particularly when large time spans are to be considered. These changes will tend to blur the standard bimodal distribution of up and down states, making it hard to separate the two states based simply on threshold.
2. Although the threshold can be automatically determined, there is a certain tendency to establish the settings manually according to the expert assessment, even when dealing with very stable recordings and well-differentiated bimodal behavior. A reliable computerized method for peak identification in the histogram of membrane potentials from recordings that are not obtained in ideal conditions could be hard to find.

An increasing amount of “non-standard” electrophysiological data (from anesthetized animals and slice recordings) and in addition long duration recordings demand automated and reliable methods for up and down states identification and characterization. We present an automatic and easy-to-use method that is able to identify and to reliably separate the two states of membrane potential, characteristic of slow wave sleep and under certain anesthesia: MAUDS (for Moving Averages for Up and Down Separation). Furthermore, the method has been engineered to be used online, in such a way that the up and down states can be visualized in real-time superimposed to the original signal, and the experiment design can include triggering events. It also provides immediate information on the statistics of the up *versus* down periods to evaluate the behavior of the network.

## Methods

### Experimental Methods

#### Slices preparation

The methods for preparing cortical slices were similar to those described previously [14]. Briefly, cortical slices were prepared from 2- to 6-month-old ferrets of either sex that were deeply anesthetized with sodium pentobarbital (40 mg/kg) and decapitated. Four hundred-micrometer-thick coronal slices of the visual cortex were cut on a vibratome. A modification of the technique developed by [36] was used to increase tissue viability. After preparation, slices were placed in an interface-style recording chamber and bathed in ACSF containing (in mM): NaCl, 124; KCl, 2.5; MgSO_4_, 2; NaHPO_4_, 1.25; CaCl_2_, 2; NaHCO_3_, 26; and dextrose, 10, and was aerated with 95% O_2_, 5% CO_2_ to a final pH of 7.4. Bath temperature was maintained at 34–35°C. Intracellular recordings were initiated after 2 hr of recovery. In order to induce spontaneous rhythmic activity, the solution was switched to ACSF containing (in mM): NaCl, 124; KCl, 3.5; MgSO_4_, 1; NaHPO_4_, 1.25; CaCl_2_, 1–1.2; NaHCO_3_, 26; and dextrose, 10.

#### Animal preparation for *in vivo* recording

Intracellular recordings *in vivo* from the primary visual cortex of cats were obtained following the methodology that we have previously described [37]. In short, adult cats were anesthetized with ketamine (12–15 mg/kg, i.m.) and xylazine (1 mg/kg, i.m.) and then mounted in a stereotaxic frame. A craniotomy (3–4 mm wide) was made overlying the representation of the area centralis of area 17. To minimize pulsation arising from the heartbeat and respiration a cisternal drainage and a bilateral pneumothorax were performed, and the animal was suspended by the rib cage to the stereotaxic frame. During recording, anesthesia was maintained with i.m. injections of both ketamine (7 mg/kg) and xylazine (0.5 mg/kg) every 20–30 min. The heart rate, expiratory CO_2_ concentration, rectal temperature, and blood O_2_ concentration were monitored throughout the experiment and maintained at 140–180 bpm, 3–4%, 37–38°C, and >95%, respectively. The EEG and the absence of reaction to noxious stimuli were regularly checked to insure an adequate depth of anesthesia. After the recording session, the animal was given a lethal injection of sodium pentobarbital. Animals were cared for and used in accordance with the Spanish regulatory laws (BOE 256; 25-10-1990) which comply with the EU guidelines on protection of vertebrates used for experimentation (Strasbourg 3/18/1986).

#### Rat barrel cortex

Adult Wistar rats (250–300 g) were used for recordings in S1 cortex. Anesthesia was induced by intraperitoneal injection of ketamine (100 mg/kg) and xylazine (8–10 mg/kg). The animals were not paralyzed. Maintenance dose of ketamine was 75 mg/kg/h. Anesthesia levels were monitored by the recording of low-frequency electroencephalogram (EEG) and the absence of reflexes. Rectal temperature was maintained at 37°C. Once in the stereotaxic apparatus, a craniotomy (2×2 mm) was made at coordinates AP –1 to −3 mm from bregma, L 4.5–6.5 mm. After opening the dura, extracellular recordings were obtained with a tungsten electrode (FHC, Bowdoinham, ME, USA). Extracellular recordings were used to adjust whisker stimulation (not shown) and to monitor the occurrence of slow oscillations. Intracellular recordings were obtained within 1 mm from the extracellular recording electrode. ***Whisker stimulation.*** A puff of air given through a 1 mm tube placed in front of the whiskers (10–15 mm) was used for stimulation. The air puff (10 ms) was controlled by a stimulator and delivered by a Picopump (WPI, Sarasota, FL). Its pressure was adjusted such that it would evoke a response that was of 50–100 µV in the extracellular recordings and between 5 and 10 mV in the intracellular recordings.

#### Recordings and stimulation

Sharp intracellular recording electrodes were formed on a Sutter Instruments (Novato, CA) P-97 micropipette puller from medium-walled glass and beveled to final resistances of 50–100 MΩ. Micropipettes were filled with 2 M potassium acetate. Recordings were digitized, acquired and analyzed using a data acquisition system (Power 1401; Cambridge Electronic Design, Cambridge, UK) and its software (*Spike* 2). Two different implementations of MAUDS where integrated in *Spike* 2: (1) using its built-in script language, and (2) as an assembler program that can be run on the sequencer included in the system. The functioning of these implementations has been tested and is further discussed in the results section. These programs, as well as MATLAB (*The MathWorks, Inc.*) implementations, are distributed as open source, and can be fetched from a web site (*http://www.geb.uma.es/mauds*), where a tutorial, examples, and a forum for MAUDS users are also available.

### Analytical Methods

The strategy we propose for characterizing up and down states in electrophysiological data is based on a method widely used in financial data analysis: crossover of moving averages.

Methods for financial time series forecasting often involve the linear transformation (averaging) of past data in order to track trends and predict trend reversals [38]. Transitions between up and down membrane regimes can be anticipated in a similar way: current and previous dynamics can predict a forthcoming change to a depolarized or hyperpolarized membrane. In the field of signal processing such systems are referred to as real-time smoothers, and its implementation is equivalent to a low-pass filtering with two cut-off frequencies.

We consider a time series of intracellular membrane potential samples. *x_i_* represents a sample in *mV* of membrane potential values. This signal is smoothed by computing for each sample a value that averages the membrane potential through a given time window.

In forecasting systems, the standard form of a moving average over the last *n* values is given at time *t* by the following expression:
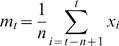
(1)


A family of implementations can be obtained when the terms in the summation are scaled according to some weighting function. One of such functions weights each value with a constant that decreases exponentially with the distance to the current value. The main property of this exponential weighting is that it gives a greater importance to recent values, while integrating over a wide temporal window. The price is a higher computational cost. This shortcoming must be taken into account when filtering physiological data recorded for a large period of time at a high sample rate. In such cases, the window size could extend along more than one hundred thousand values (2–3 s depending on the acquisition frequency). However, the implementation of exponential weighting with a first-order difference equation solves this computational problem. Equation (2) computes the exponential moving average of the last *n* values. It proceeds by combining the contribution from the previously averaged value, and the current value of the signal.
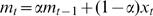
(2)where 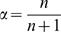, and then 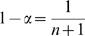 (note that α ∈ [0,1), i.e. 1 is excluded).

The recursion reduces the complexity of the original loop to an order of magnitude (two products and one addition). This expression allows the smoothing of large data vectors in real time on a conventional computer.

Higher values of *n* will expand the range of past values that influence the current value, strengthening the smoothing effect of the average. Parameter *n* is adjusted according to the dynamics of the signal. For example, in trading applications, trend tracking indicators use wide and narrow averaging windows for highly volatile and non-volatile prices, respectively.

Periods where a signal keeps its tendency to increase or decrease (trending periods) can be tracked with fitted exponential moving averages (EMAs), while changes in this trending behavior (trend reversal) is detected by crossing over two EMAs with different window sizes. In the financial world these two curves that follow the signal are generally termed short-term (or fast) and long-term (or slow) averages. For example, a short-term EMA integrates something like the last two weeks of the signal (say a commodity's price), while the long-term EMA averages the last three months. Crossings of the short-term EMA from values above the long-term curve to values below it indicate a possible change from the current trend to increase (a positive slope characteristic of buying periods) to a new decreasing period (negative slope, or selling cycle), while changes from below to above the long-term EMA indicates a change from the decreasing trend to an increasing one (negative to positive slope).

The dynamics of the electrophysiological signal that we intend to characterize depends on several factors: cortical region, level of anesthesia, depolarizing or hyperpolarizing currents, etc. While the expected frequency is about 1 Hz, in practice (including *in vitro* and *in vivo* recordings) this variable ranges between 0.2 and 1 Hz. This variability makes it necessary to adjust the method to the dynamics of each particular signal. A broad estimation of the frequency of the recorded signal suffices to compute suitable values for the window sizes of both EMAs. Expressed in seconds, the size of the windows for the slow average (*W_s_*) and the fast average (*W_f_*) are given by the following equations:
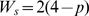
(3)

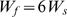
(4)where *p* is the estimated period (the inverse of the frequency) of the wave to be characterized. Here, equation (3) is defined such that the period of the wave is expected to fall below four seconds (or frequencies higher than 0.25 Hz). In a standard situation (frequency around 1 Hz) the slow EMA will be six times faster than the original signal.

The crossing points of the two EMAs are good approximations of the transitions between up and down states (i.e. of both, up and down initiation). However, some extra processing around these points can determine more precisely the onsets and offsets. The results clearly improve by analyzing the slope of the signal with a simple momentum operation. The momentum is another indicator widely used in the financial world to measure market's sentiment. It is defined as the difference between the current value of the signal and a previous value, with respect to the time difference between them. It operates, therefore, as an estimate of the slope. More precisely, equation (5) shows this relation.
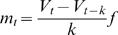
(5)where *k* is the time difference, and *f* is the sampling frequency. For example, if the membrane potential recorded at time *t* is –70 mV, and the value that was sampled 125,000 steps before was –60 mV, a frequency of 25 kHz would give a momentum of –20 mV/s, which means that around time *t* the membrane tends to hyperpolarize at a rate of some –20 mV every second.

This estimation of the slope is an indicator of the shape of the curve where the transition takes place. When the tendency to become hyperpolarized slows down at the end of an up state, we enter the flat hyperpolarized region of the down state. In terms of potential's slope, this is like moving from low (negative) values to a zero slope. The reverse is true for entering the up state: the slope increases as the membrane depolarizes. Transitions are therefore computed as the precise moments around the crossing points where the momentum raises over a certain threshold. This limit value is negative when transition is made from up to down, and positive for down to up transitions.

Finally, those excursions of the membrane potential (identified by the method as up or down states) with duration shorter than 40ms were filtered out, as in [12], [34].

The combination of these two methods (EMAs overcrossing, and a fine analysis of membrane potential around the crossing points) reliably characterizes data in ideal and noisy conditions, even in situations where the histogram-based approach might fail. In the rest of the paper the proposed method will be referred as MAUDS and its performance will be tested against the traditional method in differently shaped intracellular bistate data. Blue boxes have been used in the figures to highlight the detected up states.

## Results

Up and Down states were identified in intracellular recordings obtained from the cerebral cortex of both *in vitro* and *in vivo* preparations from different areas of the cortex (visual, prefrontal and somatosensory). In the first part of the results we describe the properties of MAUDS analyzing the recordings with the MATLAB scripts in an offline fashion. In the second part of the results we demonstrate how this method can also be used online, thus allowing to exploit the signals that it generates in order to trigger other events or to obtain immediate statistics of time distribution of up *versus* down states under different conditions. The detection of up and down states occurring in the network can also be carried out by applying MAUDS to the local field potential (LFP) (Fig. S1), detection that shows a high correlation with the one from intracellular recordings obtained simultaneously and in close vicinity to the LFP.

The characteristic shape of neuronal membrane potential during slow oscillations shows two clearly differentiated states of membrane potential: a depolarized membrane (up states) and a hyperpolarized one (down states), with relatively fast transitions between them. As said before, in short recordings, up and down states are often identified by thresholding the membrane potential. However, this method frequently fails in long recordings due to membrane potential drifting, presence of spindles, and other types of interferences like electronic noise or movement artifacts while *in vivo* (heartbeat pulsation, respiratory movements, etc). Even when the aim of the experimentalists should be to eliminate all these artifacts, we will exploit them here in order to test the robustness of the described method against other commonly used ones. Two problems have to be solved for a good characterization of the states: (1) determining the periods where depolarized (up) or hyperpolarized (down) membrane potential take place, and (2) identifying the precise points in time where these states actually start and end. As explained in the previous section, MAUDS tackles these problems with an initial broad identification of the down states by overcrossing two moving averages, and a later refinement of the initiation and termination points by a discrete processing of the membrane potential evolution in the transition interval. In general, we have observed that MAUDS performs well for any value of the long-term EMA in a wide range. On the other hand, the characterization is slightly more sensitive to the fast EMA. An optimum window size would smooth efficiently the high frequency changes of the membrane potential (isolated spikes and artifacts), being also quick enough to detect fast excursions of the signal to highly hyperpolarized regions.

**Figure 1:**
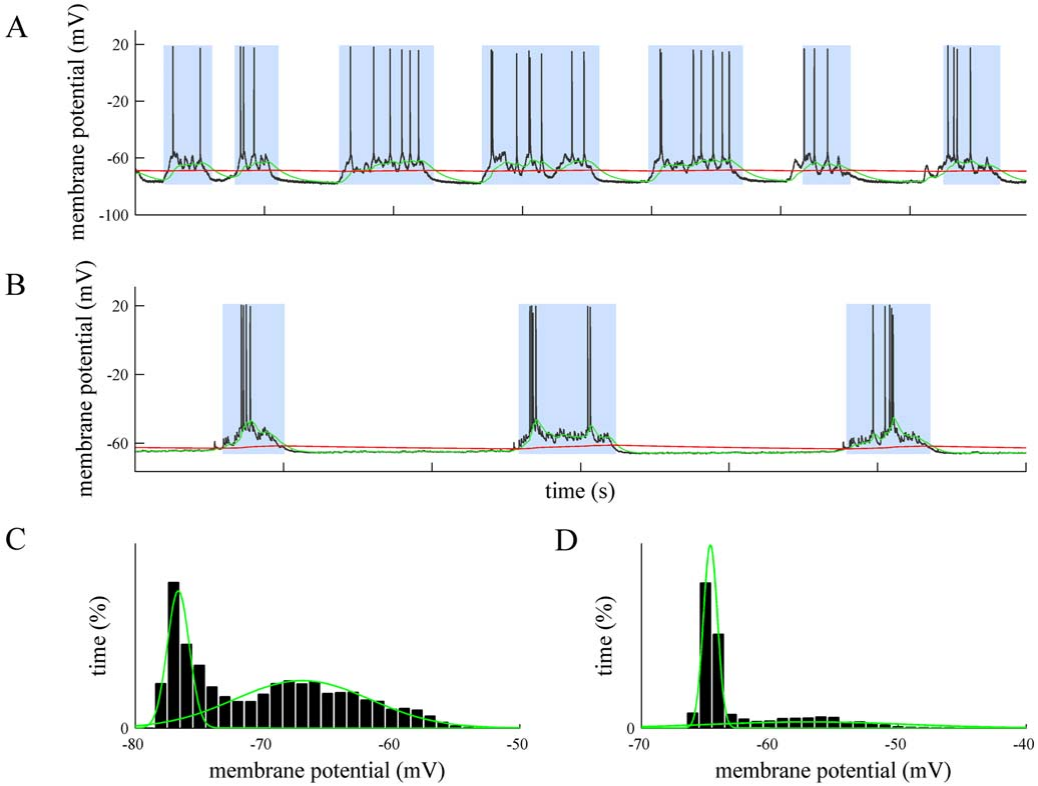
Offline separation of standard up and down states. A. Intracellular recording *in vivo* from a neuron in cat primary visual cortex. Time marks in the horizontal axes of the traces indicate 1 second interval (relative labels not shown for clarity). A fast EMA is represented as a green line and a slow EMA in red line. The points of crossing between both of them have been used to calculate the beginning and end of up states, highlighted with a blue box. Same in B. B. Intracellular recording *in vitro* from a supragranular neuron in a prefrontal cortex slice from the ferret. C. Histogram of the membrane potential values corresponding to the trace in A. It shows two clearly differentiated states separated by a transitional valley (see Gaussian fit in green superimposed to the histograms, with parameters −76.6 and −67.0 for the mean, 0.8 and 5.3 for the standard deviation). D. Histogram of the membrane potential values corresponding to the trace in B (fitting curves with parameters −64.6 and −57.0 for the mean, 0.5 and 7.6 for the standard deviation).

We studied periods of ≥ 900 seconds of intracellular fluctuations in recordings from neurons in the visual cortex of the anesthetized cat, from neurons in the somatosensorial cortex in the anesthetized rat, and from neurons recorded in oscillating ferret cortical slices obtained from prefrontal or visual cortices from the ferret (n =  20). The traces in (Fig. 1A, 1B) were recorded from two different animals and show the standard up-down behavior. These states are efficiently separated for a wide range of fast and slow EMAs. Under these recording conditions, the histograms show two different distributions of membrane potentials (Fig. 1C and D). Therefore, a simple thresholding is expected to reliably separate up and down states. (Overshadowing blue boxes show the precise limits of the up states found by MAUDS, in this and the following figures.)

**Figure 2:**
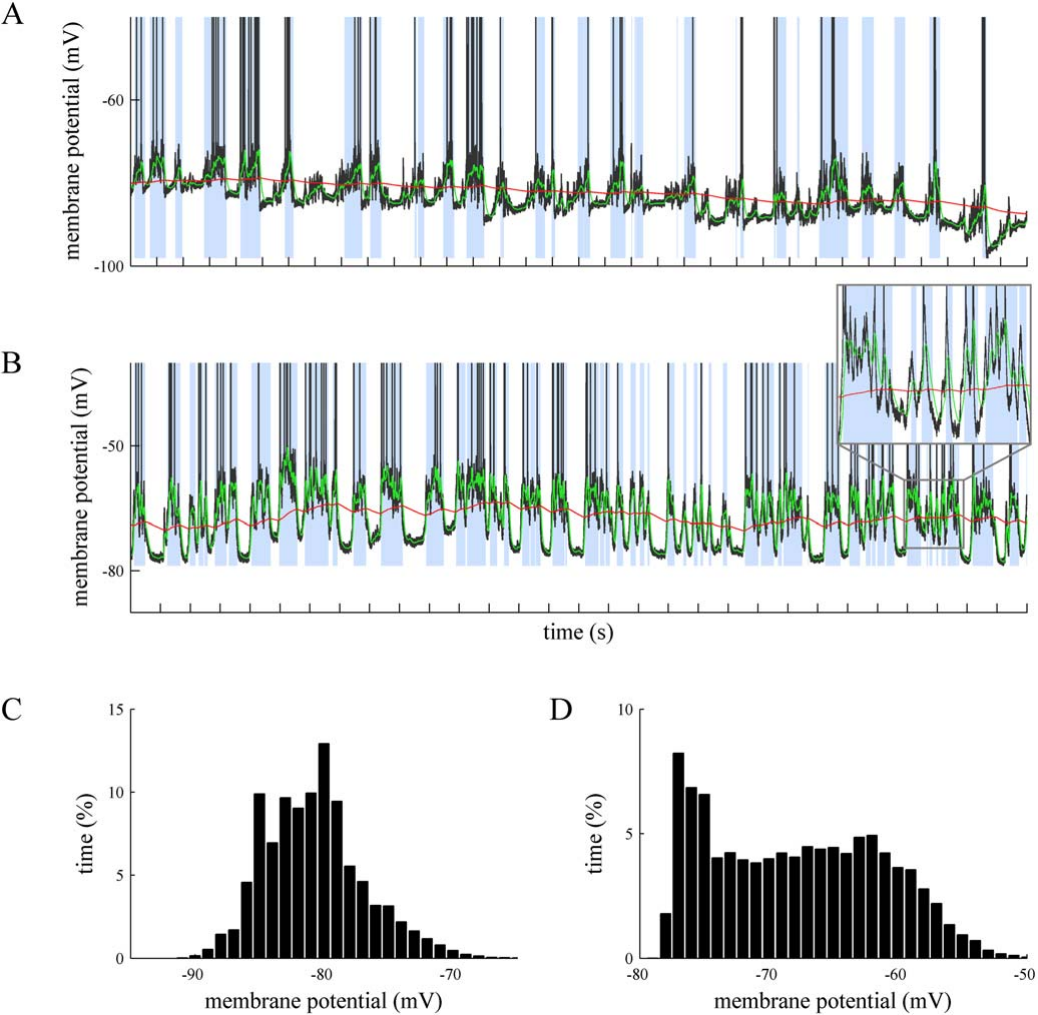
Offline up and down states separation in drifted recordings. A. *In vivo* intracellular recording from a neuron in the primary visual cortex from the cat. A drift in the membrane potential is illustrated. B. Intracellular recording *in vitro* from a neuron in the prefrontal cortex of the ferret. The slow EMA follows the average membrane potential, providing a value of reference that discriminates the up and down levels. See the high frequencies detailed in the inset. Time marks in the horizontal axes of the traces indicate 1 second interval. C and D. Histograms corresponding the A and B traces respectively. Note that in the drifted recordings the bimodality of the Vm values is not as clear as in stable recordings like in Fig. 1.

Non-standard up and down states arise when the recording scenario departs from these ideal conditions. The periodicity and homogeneity of the standard up and down states disappears, yielding either irregular fluctuations (induced for example by noise or respiration if *in vivo*), or high frequencies that blur the transitions (especially in the down states initiation). While MAUDS can still deal with these situations (traces and superimposed EMAs in Fig. 2, A and B), the resulting histogram rapidly looses the bimodal shape (Fig. 2C and D), making it harder to decide where the right threshold should be located. Since the duration of up and down states presents a large variability, it is also difficult to filter false transitions according to this feature. The histograms performed over longer recording sessions simply showed a smoothed shape, but failed to better define the two-peaks picture.

Another undesired artifact is signal drifting, caused by changes in the junction potential. In principle this effect can be prevented (chloriding silver electrodes, using an agar bridge, etc.) and compensated by commercial amplifiers, but it is usual to obtain long sequences of data where slow shifts (e.g. Fig, 2A) or fast excursions of the membrane potentials can be observed. These variations in the apparent membrane potential do not necessarily reflect any change in the current flowing through the membrane but an offset of the membrane potential value. Therefore, the bistable fluctuation of the membrane potential during rhythmic activity remains, allowing it to be studied in spite of the unstable wave it is resting on (Fig. 2).

**Figure 3:**
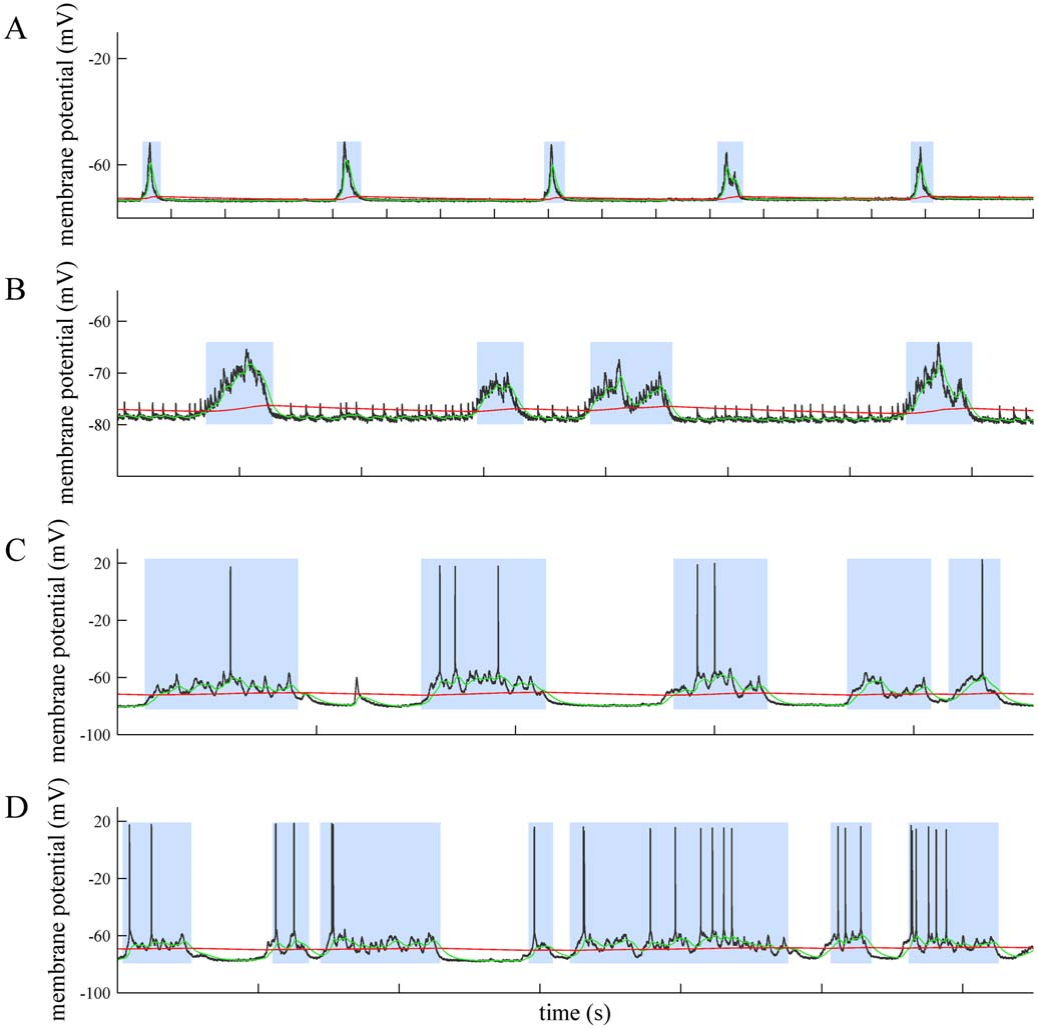
Offline detection of up and down states by MAUDS in special situations. A and B. Correctly identifying up states where no action potentials occur in highly hyperpolarized neurons recorded *in vitro* in prefrontal cortex from the ferret. Note that in B there is correct detection of down states in spite of the repetitive occurrence of short lasting sharp events. C. Filtering isolated synaptic events occurring in the middle of a down state. D. Sorting suspicious down states intermingled into long-lasting up states (third up state). C and D correspond to intracellular recordings obtained in vivo from cat's primary visual cortex. In all panels time marks in the horizontal axes of the traces indicate 1 second interval.

**Figure 4:**
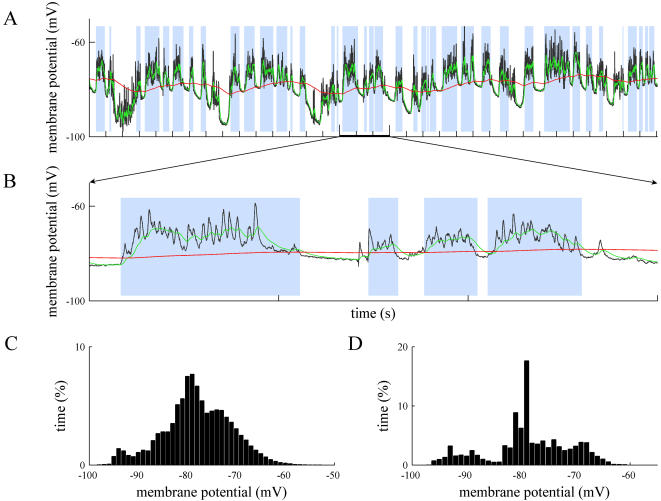
Offline identification of up and down states in intracellular recordings with artifacts. A. Intracellular recording from primary visual cortex of the cat *in vivo*. There is a respiratory movement artifact that generates rhythmic drifts of the membrane potential. B. Detail of a portion of the membrane potential shown in A (second 15, 16, 17). Time marks in the horizontal axes of the traces indicate 1 second interval. C and D. The distributions of membrane potentials in panels A and B, respectively.

In addition to drifted recordings, the proposed method correctly separates up and down states where special events take place, such as the absence of spiking activity in a hyperpolarized membrane with subthreshold oscillations (Fig. 3, A and B traces), the presence of isolated synaptic potentials (or even spikes) along well-defined down states (Fig. 3C shows a synaptic potential between the first and second up states), frustrated down state initiations that might generate misclassifications (Fig. 3D), or recordings during respiratory or other movement artifacts (Fig. 4A), where the underlying slow oscillation is still present (detailed in Fig. 4B). The histograms of membrane potential show that some bimodal distribution remains (Fig. 4D) over stable intervals, but it vanishes when applied to a few seconds interval (Fig. 4C shows the histogram for the trace on Fig. 4A).

In order to compare the performance of MAUDS with that of the histogram method, 5 recordings containing standard slow oscillation were selected (for an overall time of 145 s) and the corresponding transitions were obtained based on the histogram (best manual fitting) and with MAUDS, where a broad estimation of the oscillation frequency parameterized the slow and fast EMAs. With regard to effectivity, both methods correctly identified all the up and down states present in the recordings. On the other hand, the precision of MAUDS was compared to the histogram-based characterization according to the Coincidence Index (CoIn) described in [34]. The mean degree of overlap computed between the two series of up and down states was 91.7±0.8%, with a 97.7±1.6% CoIn for the up states, and a 85.7±2.8% for the down states. This value shows that MAUDS has a high precision in determining the transitions with respect to the traditional histogram approach.

Although the histogram method performs similarly in characterizing standard oscillations, the previous examples show that a fixed threshold will not characterize well the underlying slow oscillation in non-standard recordings. Determining the threshold for standard up and down states can easily be done in a manual way, but a criterion to deal with non-standard behavior (as in the previous examples) has not been proposed yet in the literature. For this reason, MAUDS performance can not be compared to a histogram-based characterization of non-standard slow oscillations.

In order to use MAUDS for the online analysis of intracellular recordings (Movie S1), the script was integrated in the *Spike* 2 (Cambridge Electronic Design, Ltd.) data acquisition software. As described in the Methods section, two different implementations have been coded and tested for online characterization. While the characterization of the electrophysiological signal is equivalent in both versions, the computational resources and times used differ significantly. The script version has the advantage of being coded in a high-level programming language, which is easy to understand and update by potential users. In contrast, the assembly version results extremely cryptic and is not suited for further modification by users. On the other hand, the script runs on the computer's processor, which means that it shares the resources with the recording process (that has a higher priority) resulting in characterization times that do not allow real-time triggering (around 1 s on a *Pentium* IV processor). Furthermore, the assembly language runs on the sequencer (see Methods for details), and has the advantage of a processing time that is completely independent of the computational resources, the system's load, and the recording process itself. The sequencer processes 20 instructions per millisecond, allowing a real-time interaction with the experiment: stimuli can be triggered 1 ms after the transition has been detected.

**Figure 5:**
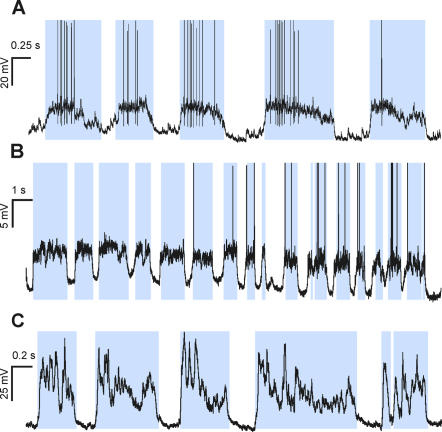
Online detection during intracellular recordings *in vivo*. A. Up states recorded in a fast spiking neuron in the primary visual cortex of the cat. B. Online detection of up and down states in an intracellular recording in cat primary visual cortex during subthreshold and suprathreshold up states in a drifted recording (note that due to the drift the suprathreshold up states seem to be more hyperpolarized than the subthreshold ones). In A and B spikes have been truncated. C. Online detection of up states recorded in the barrel cortex of a rat. In all these cases the animals were anesthetized with ketamine and xylazine (see Methods). In all panels time marks in the horizontal axes of the traces indicate 1 second interval.

**Figure 6:**
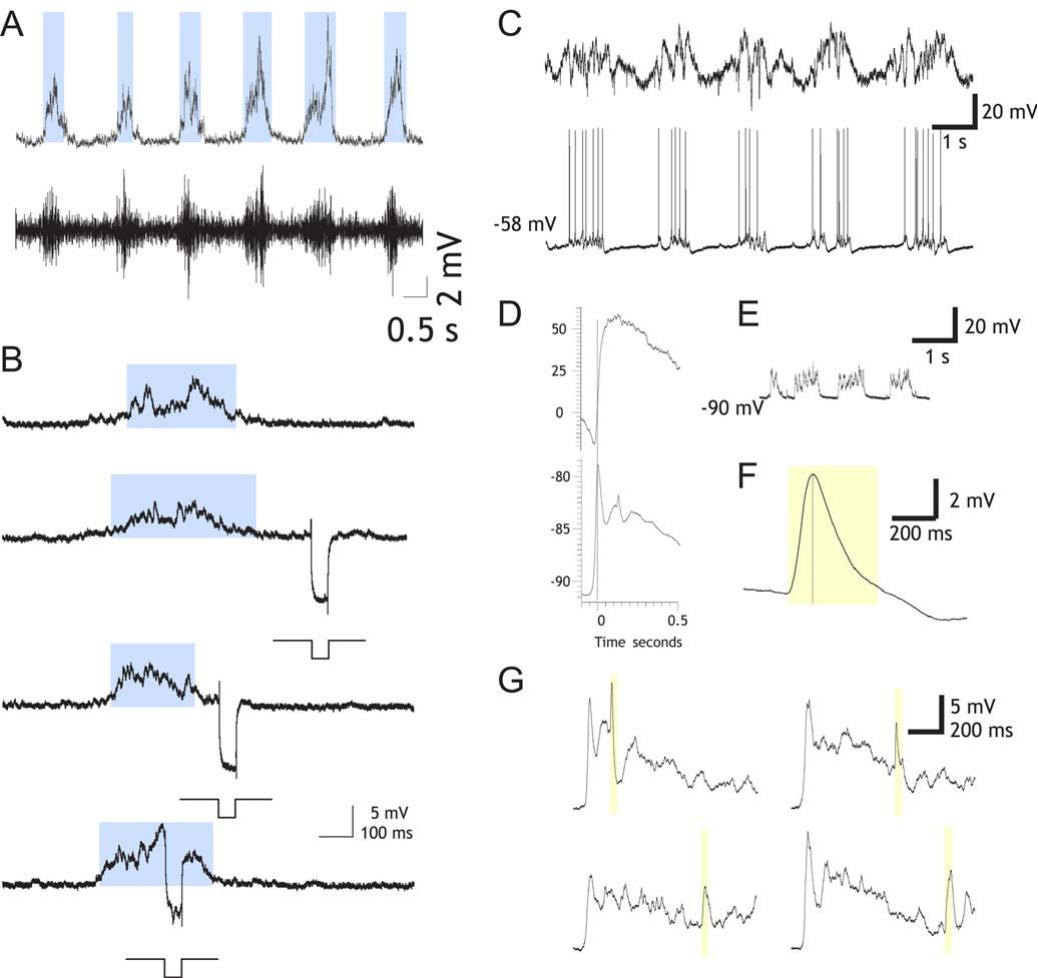
Online detection of up states and their use as triggers. A. Online detection of up states during *in vitro* intracellular recordings in primary visual cortical slices from the ferret. Bottom trace: extracellular multiunit recording representing the population firing in the vicinity from the intracellular recorded neuron. B. Online detection of up states in a recording from ferret oscillatory slices, primary visual cortex. In this case the beginning of the up state has been used to trigger a hyperpolarizing pulse (−0.2 nA) at different times with respect to the occurrence of the up state in order to estimate changes in the input resistance. C. Slow oscillations in the barrel cortex of the ketamine anesthetized rat. Unfiltered local field potential (top) and intracellular suprathreshold recording (bottom). D. Averaged up states (n = 20) using the detection of initiation of up state as a point of reference with online MAUDS analysis, LFP (top) and intracellular recording (bottom). E. Subthreshold oscillations. F. Averaged intracellular responses to a puff of air to the whiskers (n = 20). The sensory response is highlighted with a yellow box. Same in G. G. Averaged up states while giving the whisker stimulation at regular intervals after the initiation of the up state (5 in each case), four intervals are represented.

The assembly version was used to perform online characterization and pulse triggering. The detection of the transitions between up and down states was set to generate a 1-bit digital signal, differentiating the current up or down state present in the voltage recordings. This signal was recorded and used externally to trigger events by connecting it to other equipment. Online analysis of up and down states was performed in more than 40 intracellular recordings during slow oscillations occurring in the cortex of anesthetized animals *in vivo* (visual, somatosensory) and *in vitro* (visual, prefrontal). The results of the online analysis are illustrated in Figs. 5 and 6. Figure 5 represents the detection of up states during three different intracellular recording *in vivo*: supra- and subthreshold up states of different durations and amplitudes are equally detected during the recording. Identification of up and down states during recording from a fast spiking neuron (Fig. 5A) in primary visual cortex, during a drifted recording from a regular spiking cell (Fig. 5B) or subthreshold up states recorded from rat barrel cortex (Fig. 5C) are illustrated. Online analyzed drifted recordings (Fig. 5B) were still well identified. In Fig. 5B a small depolarization remained undetected. However this depolarization could hardly be defined as up states even by visual inspection and manual classification.

*In vitro* recordings were also analyzed online (Fig. 6A,B; Movie S1), and subthreshold up states are displayed, along with the population activity reflected in the multiunit recording in close vicinity of the intracellularly recorded cell. In a different neuron (Fig. 6B), the signal generated by the detection of the initiation of the up states was fed into the intracellular amplifier (*Axoclamp* 2B, *Molecular Devices Co.*) in order to generate a step of hyperpolarizing current. By regulating the delay of occurrence of the current injection, the input resistance of the neuron could be measured at different times with respect to the initiation of the up states. This signal could have been used equally for the triggering of other events of stimulation or analysis.

Online detection of up states was also used to average up states and thus determine the shape of the up state rising time, as it was done for slow oscillations recorded in the barrel cortex of the ketamine-anesthetized rat (Fig. 6 C, D). A puff of air delivered to the whiskers induced a consistent sensory response that was recorded intracellularly in the barrel cortex (Fig. 6F). The signal generated by the online detection of the up states' initiation was also used to trigger the sensory responses at particular intervals after the initiation of the up states, thus allowing systematic average of different trials (Fig. 6G).

## Discussion

Identifying the transitions between up and down cortical states is sometimes difficult and has to rely on the subjective opinion of the researcher. For example, it is not obvious when a short depolarization should be wide enough to be considered an up state or when the absence of spikes is a necessary condition to determine the presence of a down state. Understanding the cellular and network mechanisms that generate the two-state behavior generated by the cortical network therefore demands a robust and reliable method for up and down states identification. Here we have demonstrated that the traditional histogram-based approach originally described by Metherate and Ashe (1993) [22] and extensively used afterwards (e.g. [14], [19], [23]–[26], [28]–[33]), while being an efficient graphical tool for manual threshold determination under ideal conditions, lacks the adaptive computational properties to deal with fuzzy transitions, occurring during recordings that are not stable, or drifting, that develops quite often over long recordings.

Trend-following techniques of financial trading applications combined with problem-specific knowledge yields a method that robustly separates up and down states, in both ideal and fuzzy situations. This work formalizes such a method and analyses its performance in different situations characteristic of ill-defined biphasic behavior: (1) irregular shape of up and down states –variations in amplitude, frequency– (Fig. 3) (2) imprecise down state initiation, (3) signal drifting (caused by changes in the liquid junction potential at the electrode tip), or (4) artifacts due to movements during *in vivo* recordings, such as respiratory movements or heartbeat (Fig. 2).

The experiments carried out for up and down state separation show that histogram-based methods will perform well in ideal situations (as widely reported in the literature), but will fail if the signal differs from this harmonic, well-defined and non-trended behavior. On the other hand, MAUDS efficiently separates up and down states in ideal (closely fitting the best histogram-based characterization) as well as in irregular oscillation. The cases studied in this work are common in most intracellular recordings, and can be analyzed with an adaptive method of the sort of MAUDS.

Well-defined up and down states have been widely studied in the past, but how this bistate behavior departs from ideal conditions has not been reported in the literature, perhaps because of the lack of objective methods to characterize irregular situations. Such a method will allow formal quantification of these excursions, and must be based on an extended definition of the up and down states that meets conflicting experimenters' criteria. The authors believe that an algorithmic approach similar to the one presented here would definitely be a good starting point in this direction.

In order to integrate the online and offline versions, the model has been defined and tested with EMAs that compute only previous values. This is at the cost of a delay in the turning points obtained, which affects the overall performance. An offline version based on EMAs that average past and future intervals of time for each value would improve the results shown here. In spite of this delay, the predictive character of the online version has been used experimentally to trigger stimuli and to manipulate cell membrane voltage at specific times along the oscillation. This is of great interest for experimentalists to study the impact of up and down states on signal processing (e.g. changes in conductance or in synaptic transmission and plasticity). Exponential weighting has proved to perform well, since it reacts faster, minimizing the lag between the predictive moving average and the actual data. The method is also expected to perform well in this type of interactive experiments, since the presence of sensory stimuli, current injection, or other manipulations interspersed with the oscillation will not interfere with the turning points. Only the presence of short down states might be problematic, since the artifacts might cut them. The general approach exposed here would be easily fitted to the conditions of particular experimental settings.

The method formalized in this paper has been coded as a Spike 2 script, an assembly program, and also embedded in a MATLAB toolbox. All these programs are available online as an open-source code. The MATLAB implementation exploits fast matrix operations and the powerful graphical capabilities of this programming language, and can analyze electrophysiological raw data formatted as ASCII or MATLAB binary files. The code has been optimized and computes more than a million membrane potential samples per second on a PIV 2.8GHz with 0.5GB memory (this computer processes a file containing 10 minutes of intracellular membrane potential sampled at 25 kHz in some 13 seconds). On the other hand, the Spike 2 implementations are designed for online data processing, allowing real-time characterization and visualization (script version), and triggering of stimuli (sequencer version).

Further work has to be done in order to improve two different aspects of MAUDS: (1) the adaptive capabilities of the proposed method, by automatically setting the window size of the fast EMA, that can be done based on local membrane potential variability, or exploring ranges of values where the separation remains stable; and (2) a complete validation of MAUDS over an extensive set of intracellular and extracellular data (Fig. S1) recorded in different cortical areas. While the authors expect a good general performance, even with minor changes in the parameter set, the forum set up in the MAUDS website is expected to feedback about this question, as more experimenters report on the application of MAUDS to recorded datasets.

## Supporting Information

Figure S1 MAUDS detection of up and down states on the Local Field Potential recording and comparison with detection in the intracellular recording. Intracellular (A) and LFP (B) simultaneous recording in the rat barrel cortex. LFP was recorded unfiltered. MAUDS analysis has been applied off-line to both channels independently. Blue boxes highlight the detected up states in each of the recordings. Applying the concept of Coincidence Index (CoIn) described in (Mukovski et al. Cerebral Cortex 17:400, 2007), computed CoIn between both channels was 85.7%, with a 89.3% CoIn for the up states and a 82.1% for the down states.
(0.55 MB TIF)

Movie S1 Online detection of up and down states applying MAUDS to the intracellular recordings. Slow rhythm recorded in the barrel cortex of an anesthetized rat. Top panel: Online up states detection (trace going up), Middle panel: Unfiltered LFP. Bottom panel: Intracellular recording.
(2.23 MB SWF)
