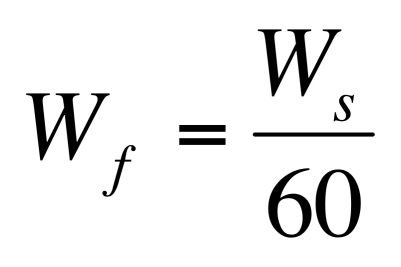# Correction: Robust Off- and Online Separation of Intracellularly Recorded Up and Down Cortical States

**DOI:** 10.1371/annotation/90ea969c-7ada-4538-aa24-17878562b995

**Published:** 2009-07-28

**Authors:** Yamina Seamari, José A. Narváez, Francisco J. Vico, Daniel Lobo, Maria V. Sanchez-Vives

In the Methods subsection "Analytical Methods," Equation 4 is incorrect. Please view the correct equation here: