# Supplementary figures and images for: Robust Off- and Online Separation of Intracellularly Recorded Up and Down Cortical States
Source: PLoS One. 2007 Sep 12;2(9):e888. doi: 10.1371/journal.pone.0000888 (PMC1964538; doi:10.1371/journal.pone.0000888)

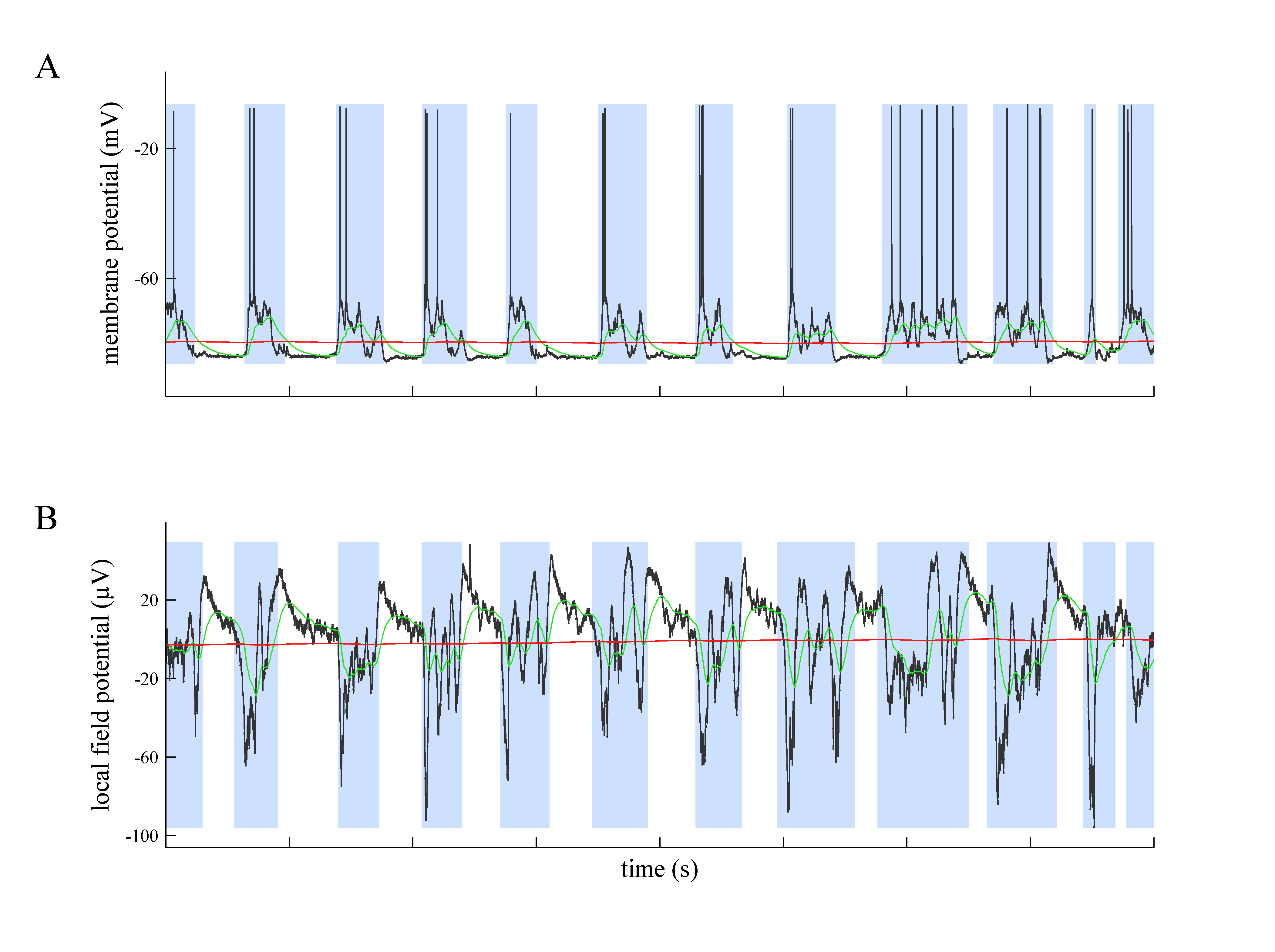

Supplement: Figure S1 — MAUDS detection of up and down states on the Local Field Potential recording and comparison with detection in the intracellular recording. Intracellular (A) and LFP (B) simultaneous recording in the rat barrel cortex. LFP was recorded unfiltered. MAUDS analysis has been applied off-line to both channels independently. Blue boxes highlight the detected up states in each of the recordings. Applying the concept of Coincidence Index (CoIn) described in (Mukovski et al. Cerebral Cortex 17:400, 2007), computed CoIn between both channels was 85.7%, with a 89.3% CoIn for the up states and a 82.1% for the down states. (0.55 MB TIF) [file pone.0000888.s001.tif]
